# Supplementary figures and images for: Shaping Neural Circuits by High Order Synaptic Interactions
Source: PLoS Comput Biol. 2016 Aug 12;12(8):e1005056. doi: 10.1371/journal.pcbi.1005056 (PMC4982676; doi:10.1371/journal.pcbi.1005056)

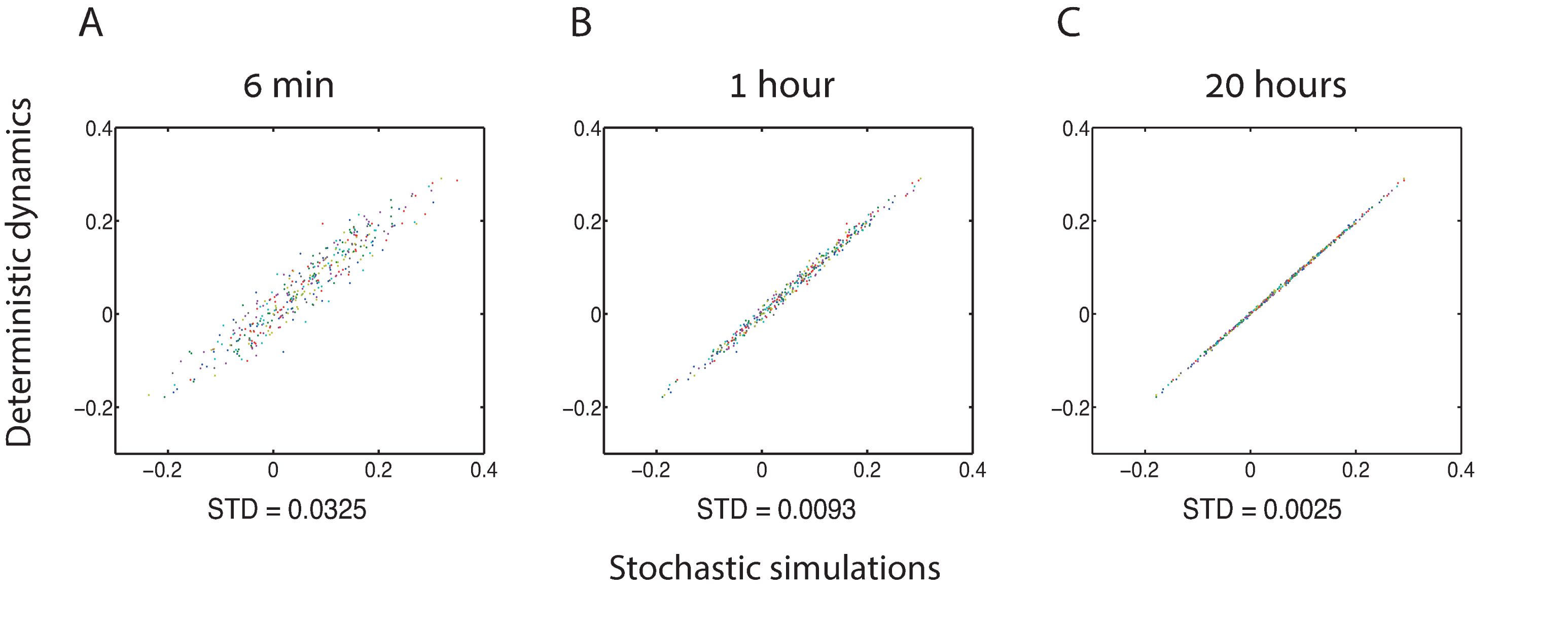

Supplement: S1 Fig — Scatter plot of the analytical expression for the average change in the synaptic efficacy (vertical axis), against the change in the synaptic efficacy generated by STDP (Eqs 6–8) in a stochastic simulation of stochastic spiking neurons, averaged over 6 minutes (A), 1 hour (B), and 20 hours (C). Each point represents the change in one synapse, where all synapses belong to the same connectivity matrix. The standard deviation (STD) listed under each panel is the mean square distance, across all synaptic pairs, between the change of the synaptic efficacy in the stochastic simulation and the prediction of the deterministic theory. The synaptic efficacies are drawn independently from a uniform distribution between [0,2NWmax], where Wmax = 0.9, N = 20, and are kept fixed during the stochastic simulations. (TIF) [file pcbi.1005056.s001.tif]

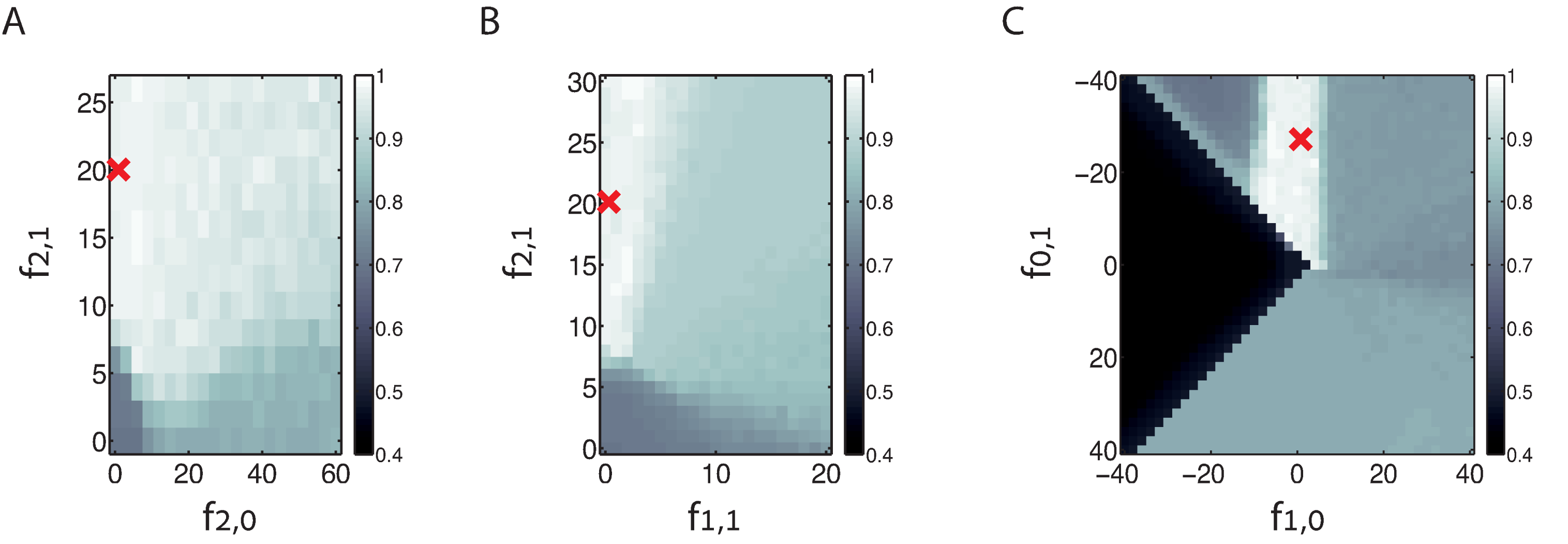

Supplement: S2 Fig — A. Chain score as a function of the motif coefficients f2,0 (horizontal axis) and f2,1 (vertical axis). B. Chain score as a function of the motif coefficients f1,1 (horizontal axis) and f2,1 (vertical axis). The simulations in A and B include also a contribution from the motif {0, 1} with fixed f0,1, and f1,2 = −f2,1. C. Reproduction of Fig 6A. The red cross designates a set of motif coefficients identical to those marked by red crosses in panels A-B. In all panels, each data point represents an average over ten simulations, each with a different realization of the initial random connectivity. (TIF) [file pcbi.1005056.s002.tif]

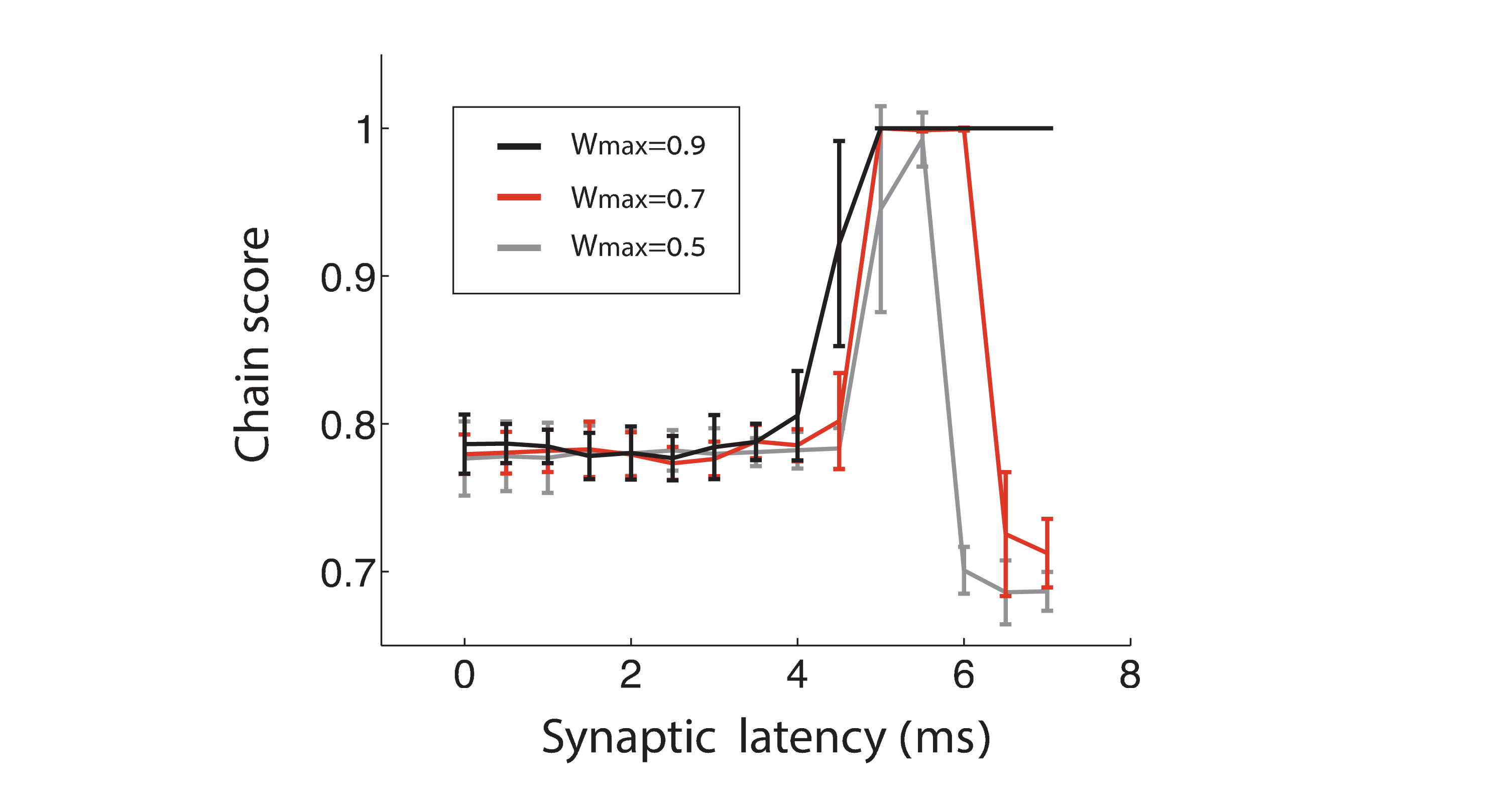

Supplement: S3 Fig — Chain score of the steady state connectivity, obtained from simulations of the complete dynamics (Eq 2). Horizontal axis: synaptic latency. Each line corresponds to simulations with a different choice of Wmax: 0.9 (black), 0.7 (red), 0.5 (gray). Each data point represents an average over ten simulations, each with a different realization of the initial random connectivity, and the error bars represent the standard deviation of the chain score. All other parameters are specified in Methods. (TIF) [file pcbi.1005056.s003.tif]

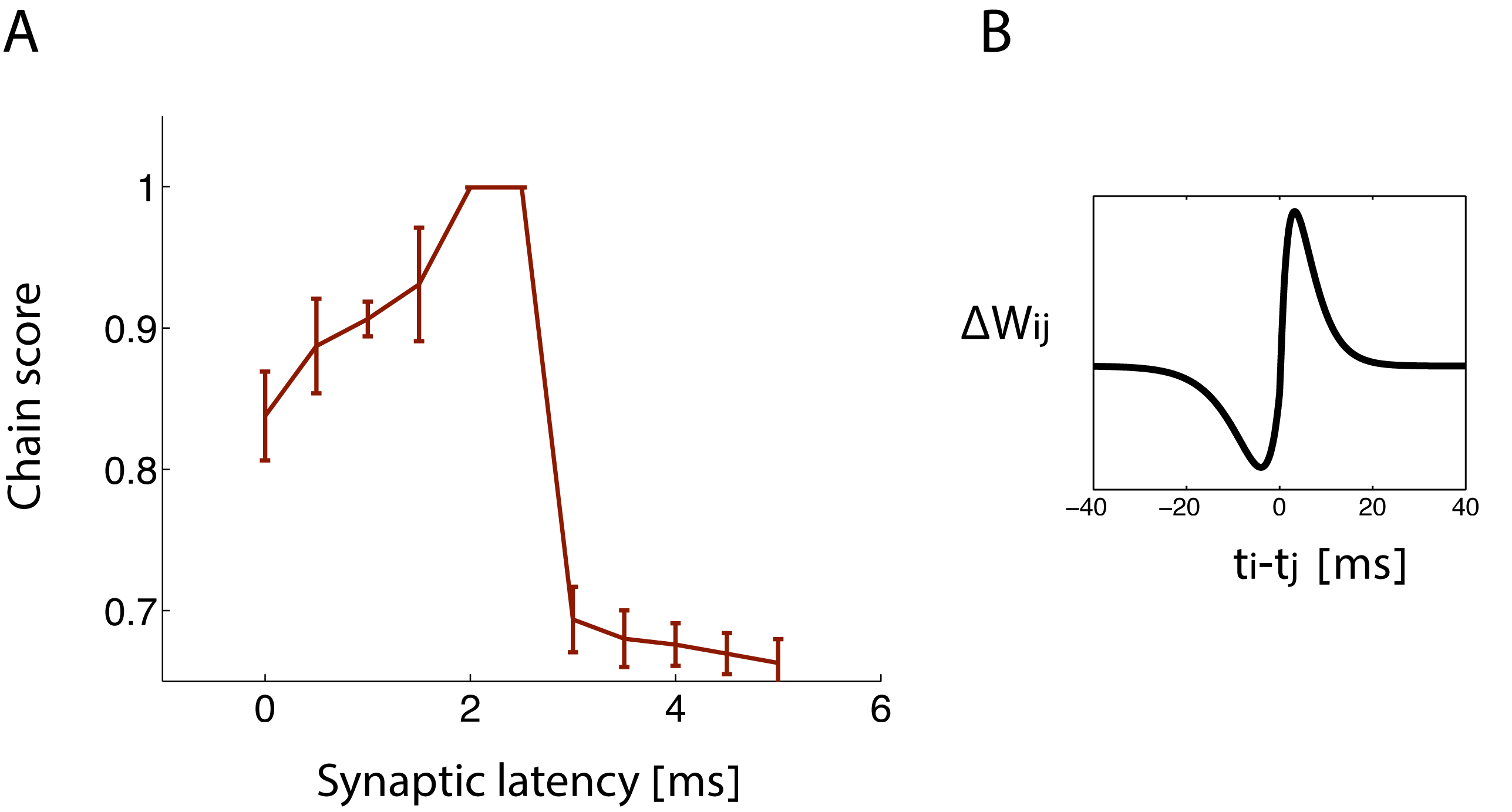

Supplement: S4 Fig — A. Chain score of the steady state connectivity, obtained from simulations of the complete dynamics (Eq 2) with a non antisymmetric STDP function (see Methods). Horizontal axis: synaptic latency. Each data point represents an average over ten simulations, each with a different realization of the initial random connectivity. B. An illustration of the non antisymmetric STDP function. (TIF) [file pcbi.1005056.s004.tif]

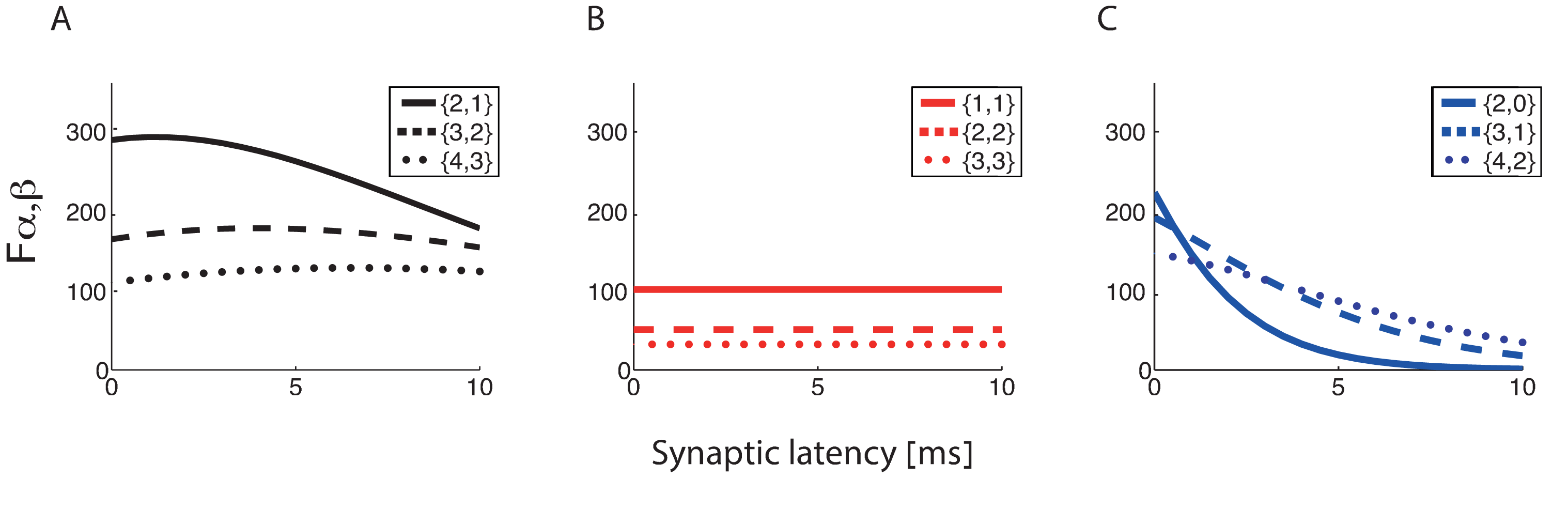

Supplement: S5 Fig — A. Contribution of motifs with α − β = 1 weakly depends on the synaptic latency. B. Contribution of motifs with α = β does not depend on the synaptic latency. C. Contribution of motifs with α − β = 2 decays rapidly as a function of the synaptic latency. All the parameters are specified in Methods. (TIF) [file pcbi.1005056.s005.tif]
